# Impact of cytokine release syndrome on cardiac function following CD19 CAR-T cell therapy in children and young adults with hematological malignancies

**DOI:** 10.1136/jitc-2020-001159

**Published:** 2020-09-03

**Authors:** Haneen Shalabi, Vandana Sachdev, Amita Kulshreshtha, Julia W Cohen, Bonnie Yates, Doug R Rosing, Stanislav Sidenko, Cindy Delbrook, Crystal Mackall, Brandon Wiley, Daniel W Lee, Nirali N Shah

**Affiliations:** 1 National Cancer Institute/Pediatric Oncology Branch, National Institutes of Health, Bethesda, Maryland, USA; 2 National Heart, Lung, and Blood Institute/Cardiovascular Branch, National Institutes of Health, Bethesda, Maryland, USA; 3 Department of Pediatrics, Center for Cancer Cell Therapy, Stanford Cancer Institute, Stanford University, Stanford, California, United States; 4 Critical Care Medicine Department, National Institutes of Health, Bethesda, Maryland, USA; 5 Minneapolis Heart Institute, Minneapolis, Minnesota, USA; 6 Division of Pediatric Hematology/Oncology, University of Virginia, Charlottesville, Virginia, USA

**Keywords:** receptors, chimeric antigen, cytokines, hematologic neoplasms, immunotherapy, pediatrics

## Abstract

**Background:**

Chimeric antigen receptor (CAR) T-cell-associated cytokine release syndrome (CRS) may present with tachycardia, hemodynamic instability and reduced cardiac function. Pediatric CAR studies examining cardiac toxicity are limited.

**Methods:**

We report on cardiac toxicity observed in children and young adults with hematologic malignancies enrolled in a CD19-28ζ CAR T-cell phase I trial (NCT01593696). All patients had a formal baseline echocardiogram. Real-time studies included echocardiograms on intensive care unit (ICU) transfer, and serial troponin and pro-B-type natriuretic peptide (pro-BNP) in the select patients.

**Results:**

From July 2012 to March 2016, 52 patients, with a median age of 13.4 years (range 4.2–30.3) were treated. CRS developed in 37/52 (71%), which was grade 3–4 CRS in nine patients (17%). The median prior anthracycline exposure was 205 mg/m^2^ (range 70–620 mg/m^2^) in doxorubicin equivalents. The median baseline left ventricle ejection fraction (LVEF) and baseline LV global longitudinal strain (GLS) were 60% (range 50%–70%) and 16.8% (range 14.1%–23.5%, n=37) respectively. The majority, 78% (29/37), of patients had a reduced GLS <19% at baseline, and 6% (3/52) of patients had baseline LVEF <53%. ICU transfers occurred in 21 patients, with nine requiring vasoactive hemodynamic support and three necessitating >1 vasopressor. Six (12%) patients developed cardiac dysfunction (defined by >10% absolute decrease in LVEF or new-onset grade 2 or higher LV dysfunction, per CTCAE v4), among whom 4 had grade 3–4 CRS. Troponin elevations were seen in 4 of 13 patients, all of whom had low LVEF. Pro-BNP was elevated from baseline in 6/7 patients at the onset of CRS, with higher levels correlating with more severe CRS. Cardiac dysfunction fully resolved in all but two patients by day 28 post-CAR.

**Conclusion:**

Cardiac toxicity related to CD19-28ζ CAR T-cell-associated CRS was generally reversible by day 28 postinfusion. Implementation of more frequent monitoring with formal echocardiograms incorporating systemic analysis of changes in GLS, and cardiac biomarkers (troponin and proBNP) may help to earlier identify those patients at highest risk of severe cardiac systolic dysfunction, facilitating earlier interventions for CRS to potentially mitigate acute cardiac toxicity.

## Introduction

Chimeric antigen receptor (CAR)-modified T-cells targeting CD19 have proven benefit for the treatment of multiply relapsed or refractory CD19+ acute lymphoblastic leukemia (ALL).^1–9^ Tisagenlecleucel, a CD19/4-1BB CAR T-cell product, received US Food and Drug Administration) approval in 2017 for pediatric and young adult patients with B-cell ALL that is refractory or in second or greater relapse. The application of CAR T-cells for the treatment of ALL continues to broaden.^10^

Cytokine release syndrome (CRS), a systemic inflammatory response syndrome due to supraphysiological immune system activation, is recognized as a potentially life-threatening adverse effect of CAR T-cell therapy.^1 3 10–15^ While mild CRS can be self-limited, manifesting with flu-like symptoms and fever, severe CRS can be marked by hypotension, hypoxia and end-organ dysfunction.^10 16^ Cardiovascular dysfunction is a significant and yet incompletely defined aspect of CRS. The hemodynamic abnormalities associated with CRS can range from tachycardia to life-threatening hypotension.

The dynamics of CRS-associated cardiac dysfunction are not well established^17 18^ and there is a paucity of available information, particularly, in children.^19^ One anti-CD19 CAR T-cell trial documented shock requiring vasoactive infusion in 13.6% of participants.^20^ Experiences to date suggest that clinically significant hypotension due to CRS (grades 3–4) occurs in 22%–38% of patients receiving CAR T-cell therapy.^3 6 9^ Based on a single-institution review of pediatric patients, predictors of hypotension requiring inotropic support include patients with pretreatment leukemic blast count >25% or pre-existing cardiac dysfunction.^21^ Considering the patient population receiving CAR T-cell therapy may have pre-existing cardiac dysfunction due to prior anthracycline therapy and/or radiation exposure, we recognize that this population may be particularly vulnerable to cardiac complications. It is important to gain a more comprehensive understanding of the cardiovascular side effect profile of CRS in an effort to understand risk factors and optimize management strategies for its associated cardiotoxicity.^22^

In this report, we systematically review the experience of cardiac toxicity seen with CD19-28ζ CAR T-cell therapy for pediatric and young adult patients receiving this investigational therapy at the National Cancer Institute. Limited real-time cardiac monitoring was combined with a retrospective analysis of baseline cardiac profiles and risk factors within this patient population. Specifically, we evaluated the association between severity of CRS and the development of cardiac dysfunction. We additionally explored the feasibility and utility of serial cardiac biomarker testing (troponin and pro-B-type natriuretic peptide (proBNP)) as an indicator of myocardial injury and cardiac dysfunction in the setting of CD19 CAR T-cell therapy.

## Methods

In this retrospective analysis, we systematically evaluated cardiac toxicity in patients treated with autologous T-cells engineered to express a CD19 CAR incorporating an anti-CD19 single-chain variable fragment plus T-cell receptor ζ and CD28 signaling domains in a phase I clinical trial approved by the National Cancer Institute Institutional Review Board (NCT01593696). Eligible patients were aged 1–30 years with CD19+ B ALL or non-Hodgkins lymphoma, relapsed or refractory to standard therapy plus at least one salvage regimen and were treated between July 2012 and May 2016. Per protocol, all patients had to be >14 days from systemic chemotherapy (including anthracyclines) and must have recovered from the acute side effects of their prior therapy.

The following pretreatment characteristics were abstracted from medical records: oncologic diagnosis, Lansky/Karnofsky performance status, bone marrow blast percentage, central nervous system disease status, extramedullary disease status, previous treatment (chemotherapy, immunotherapy, radiation therapy and hematopoietic stem cell transplant (HSCT)), cumulative anthracycline dose, and any prior receipt of dexrazoxane (if available). In addition, the lymphodepleting chemotherapy regimen, CAR T-cell dose, CRS grade and any required treatment for management of CRS (tocilizumab, corticosteroids) were recorded. CRS was graded using Lee criteria,^13^ and ASTCT consensus CRS grading was retrospectively incorporated.^23^ Other details about the clinical course during CRS, including hypotension-requiring vasoactive support and requirement for mechanical ventilation, were also obtained.

All patients had formal baseline transthoracic echocardiograms performed using commercially available systems with measurements performed according to American Society of Echocardiography guidelines.^24^ The left ventricular ejection fraction (LVEF) was calculated using the biplane Simpson’s formula (normal ≥53%). During the trial, real-time monitoring including cardiology consultation, echocardiograms, and serial cardiac evaluations (specifically troponin and pro-BNP assays) based on clinical status were performed in a limited subset of patients after the non-fatal cardiac arrest of patient 14.^6^ Best practice guidelines were used depending on the clinical scenario.

Global longitudinal strain (GLS), a measure of myocardial deformation used to detect subclinical LV dysfunction, was measured retrospectively on previously performed echocardiograms using specialized strain software (EchoInsight, Epsilon Imaging, Ann Arbor, Michigan, USA). Strain values were compared with reference ranges provided in a recent meta-analysis.^25^ The GLS data are expressed as absolute value (|%|). Formal follow-up echocardiogram reports, troponin and pro-BNP levels were available in a subset of patients. Tachycardia was defined by >100 beats per minute in patients>18 years old and adjusted for baseline pediatric normal values in those <18 years old.^26^

The primary endpoint was cardiac dysfunction defined as a>10% absolute decrease in LVEF compared with baseline or new-onset LV systolic dysfunction (grade 2, LVEF <50%). Severe cardiac dysfunction was defined by new onset LV systolic dysfunction >grade 3 or LVEF <40%), using CTCAE V.4.0.^27^ On trial,>grade 3 hypotension (sustained >24 hours of therapy or hypotension requiring vasopressor support) was captured. Elevated troponin levels above normal values and elevation in pro-BNP above patients’ baseline were secondary endpoints. All patients who were treated were included in the data extraction and analysis, with impact of CRS on cardiac toxicity limited to those who had CRS.

### Statistical methods

Enrollment characteristics were analyzed as descriptive variables, with summary statistics calculated. Baseline characteristics were summarized as number and percentage of patients for categorical variables and median and range for continuous variables. We compared the distribution of the paired outcome variable LVEF and proBNP between pre-CAR and post-CAR using Wilcoxon rank sum test. Post-CAR nadir LVEF and peak proBNP values were used for assessment. Fisher’s exact tests was used to compare outcomes between two categorial variables comparing those with and without cardiac dysfunction. Mann-Whitney U tests were used to compare unpaired data sets. Statistical analyzes were performed using Prism software, with a two-tailed p*<*0.05 used to assess for statistical significance.

## Results

### All patient characteristics (n=52)

Baseline characteristics for the 52 patients treated with CD19 CAR T-cells are shown in table 1. The median age was 13.4 years (range 4–30). Fifty patients had ALL, 2 had non-Hodgkin’s lymphoma. Thirty-nine patients (75%) had multiply relapsed disease, while 13 patients (25%) had primary refractory disease. Twenty-three patients (44%) had undergone at least one prior allogeneic total body irradiation (TBI)-based HSCT, and 11 patients (21%) had undergone prior immunotherapy. The median Lansky/Karnofsky performance status was 90% (range 40%–100%). All patients had prior anthracycline exposure, with a median of 205 mg/m^2^ (range 70–620). The median baseline LVEF was 60% (range 50–70), and 3 patients had abnormal LVEF <53% at the time of study enrollment. GLS data was retrospectively analyzed on previously performed echocardiograms in 37 patients. Fifteen patients did not have images of adequate quality and frame rate to calculate baseline GLS data (2 studies were performed at an outside hospital, 13 studies from our hospital were technically difficult). Median baseline LV GLS was 16.8% (range: 14.1%–23.5%, n=37), with 78% (29/37) of patients having a reduced GLS (<19%) at baseline.^28^

**Table 1:** Patient demographics

		All treated subjects n=52	Cardiac dysfunction n=6	No cardiac dysfunction n=46	P value*
Age, median (range), year		13 (4–30)	18 (10–30)	13 (4–27)	0.059
Male, n (%)		41 (78.8)	6 (100)	35 (76.1)	0.32
Diagnosis	ALL, n (%)	50 (96.1)	5 (83.3)	45 (97.8)	0.22
	NHL, n (%)	2 (3.8)	1 (16.7)	1 (2.2)	
Primary refractory		13 (25)	3 (50)	10 (21.7)	0.34
Prior lines of therapy	>4, n (%)	9 (17.3)	0 (0)	9 (19.6)	
	2–4, n (%)	43 (82.7)	6 (100)	37 (80.4)	
Prior HSCT	0, n (%)	29 (55.8)	3 (50)	26 (56.5)	1.00
	1, n (%)	18 (34.6)	3 (50)	15 (32.6)	
	2, n (%)	5 (9.6)	0 (0)	5 (10.9)	
Prior TBI, n (%)		23 (44)	3 (50)	20 (43.4)	1.00
Prior immunotherapy, n (%)		11 (21.1)	1 (16.7)	10 (21.7)	1.00
Prior anthracycline exposure, median (range), doxorubicin equivalents		205 (70–620)	275 (110–571)	205 (70–620)	0.23
Baseline left ventricular ejection fraction, median (range), %		60 (50–70)	61 (50–70)	60 (50–72)	0.59
Baseline left ventricular global strain, median (range), % n=37		16.8 (11.6 to 23.5)	14.4 (11.6 to 18.7)†	17 (14.1 to 23.5)	0.04
Performance status, median (range) %		90 (40–100)	80 (40–90)	90 (50–100)	0.07

Cardiac dysfunction is defined as a >10% absolute decrease in left ventricular ejection fraction (LVEF) compared with baseline or new onset left ventricular systolic dysfunction >grade 2, LVEF <50%. Any biological therapy used to treat cancer, for example, CAR T cells, antibody-based therapy.

*The p value is comparing baseline characteristics of those with and without cardiac dysfunction. Global longitudinal strain (GLS) is presented in absolute numbers (%).

†Only four patients had baseline GLS measured.

ALL, acute lymphoblastic leukemia; CAR, chimeric antigen receptor; HSCT, Hematopoietic stem cell transplant; NHL, non-Hodgkin's Lymphoma; TBI, total body irradiation.

### CRS characteristics (n=37)

Restricted to the 37 patients who developed CRS, characteristics and outcomes are described below and are shown in table 2. The median time to onset of CRS was 5 days (range 1–12), with 28 of 37 patients (76%) developing grades 1–2 CRS, and 9 patients (24%) developing grades 3–4 CRS. Seven patients with grade 3 or 4 CRS received tocilizumab, 4 of whom also received concurrent steroids. Thirty-three patients with CRS had at least one formal echocardiogram performed post-infusion, 19 of whom had echocardiograms done during CRS at a median time of 6 days (range 2–12) postinfusion.

**Table 2:** Characteristics of patients with cytokine release syndrome (CRS)

		Subjects with CRS n=37	Cardiac dysfunction n=6	No cardiac dysfunction n=31	P value*
Time to onset of CRS, median (range), d		5 (1–12)	2.5 (1–5)	5.5 (1–12)	0.015
CRS max grade†	1, n (%)	14 (37.8)	0	14 (45.1)	0.022‡
	2, n (%)	14 (37.8)	2 (33)	12 (38.7)	
	3, n (%)	6 (16.2)	2 (33)	4 (12.9)	
	4, n (%)	3 (8.1)	2 (33)	1 (3.2)	
ASTCT CRS Max Grade§	1, n (%)	16 (43.2)	0	16 (51.6)	0.0004†
	2, n (%)	9 (24.3)	0	9 (29.0)	
	3, n (%)	9 (24.3)	4 (66.7)	5 (16.1)	
	4, n (%)	3 (8.1)	2 (33.3)	1 (3.2)	
Duration of fever >38, median (range), d		5 (1–14)	5 (4–8)	5 (1–14)	0.67
Duration of fever >40, median (range), d		3 (1–6)	4 (3–6)	2.5 (1–6)	0.059
Duration of tachycardia¶, median (range), d		6 (1–30)	8 (6–9)	5 (1–30)	0.10
ICU transfer, n (%)		21 (56.8)	6 (100)	15 (48.4)	0.026
Received Tocilizumab, n (%)		7 (18.9)	4 (66)	3 (9.7)	0.006
Received steroids, n (%)		4 (10.8)	3 (50)	1 (3.2)	0.11
Required vasopressor support	One agent	6 (16.2)	2 (33)	4 (12.9)	0.14**
	>1 agent	3 (8.1)	1 (16.7)	2 (6.5)	
Required milrinone, n (%)		1 (2.7)	1 (16.7)	0	0.16
Required mechanical ventilation, n (%)		4 (10.8)	3 (50)	1 (3.2)	0.009

Cardiac dysfunction is defined as a >10% absolute decrease in left ventricular ejection fraction (LVEF) compared with baseline or new-onset left ventricular systolic dysfunction >grade 2, LVEF <50%.

*The p value is comparing treatment course characteristics of those with and without cardiac dysfunction.

†CRS max grade as per Lee *et al* Blood 2014.

‡CRS grading compared mild CRS, grade 1/2 vs severe CRS grade 3/4.

§ASTCT CRS grading was retrospectively incorporated.

¶n=36.

**P value compared requiring any pressor support versus none in those with and without cardiac dysfunction.

ASTCT, American Society for Transplantation and Cellular Therapy; ICU, Intensive care unit.

Thirty-six patients developed sinus tachycardia of a median duration of five consecutive days (range 1–30 days). Twenty-one patients (57%) who had CRS required transfer to the intensive care unit (ICU), nine patients (24%) developed hypotension-requiring vasopressor support, with three patients requiring two or more vasoactive medications for hypotension. All patients who required vasoactive medications received norepinephrine as a first line agent, with the addition of vasopressin (n=3) and epinephrine (n=1) as second-line and third-line agents, respectively. The median peak dose of norepinephrine received in these patients was 8 μg/kg/min, (range 0.15–45 μg/kg/min). One patient, previously described^6^ and further outlined below, developed reversible cardiac arrest, and was the only patient who required milrinone for cardiac support. The median time to initiation of vasopressor support was 6 days post-CAR T-cell infusion (range 3–8 days), and the median duration of vasopressor support was 3 days, (range 1–7 days). Three of nine patients requiring vasopressor support developed new cardiac dysfunction during their CRS course, with findings of LV systolic dysfunction (table 3).

**Table 3:** Characteristics of patients with cardiac dysfunction

Patient	Prior anthracycline exposure (mg/m^2^)	Baseline LVEF, %	Lowest LVEF, %	Max CRS* grade	Vasoactive support?	Troponin elevation?	Peak troponin (ng/mL)
14	360	64	20	4	Yes	Yes	6.23
16	110	70	45	4	No	Yes	0.117
39	195	60	25	3	No	Yes	0.113
45	570	55	10	4	Yes	No†	<0.010
51	355	50	40	2	No	No†	<0.010
52	180	61	45	2	Yes	Yes	0.016

Cardiac dysfunction is defined as a >10% absolute decrease in left ventricular ejection fraction (LVEF) compared with baseline or new onset left ventricular systolic dysfunction >grade 2, LVEF <50%.

*CRS max grade as per Lee *et al*, Blood 2014.

†Troponin levels were drawn and undetectable, lower limit of detection 0.010 ng/mL.

CRS, cytokine release syndrome.

No clinically apparent cardiac dysfunction occurred in the 15 patients who did not develop CRS; 13/15 patients had postinfusion formal echocardiograms performed at a median of 25 days (range 7–47) postinfusion. All echocardiograms demonstrated stable cardiac function as compared with the preinfusion echocardiogram.

### Cardiac dysfunction post-CAR T-cell therapy (n=6)

**Figure 1:**
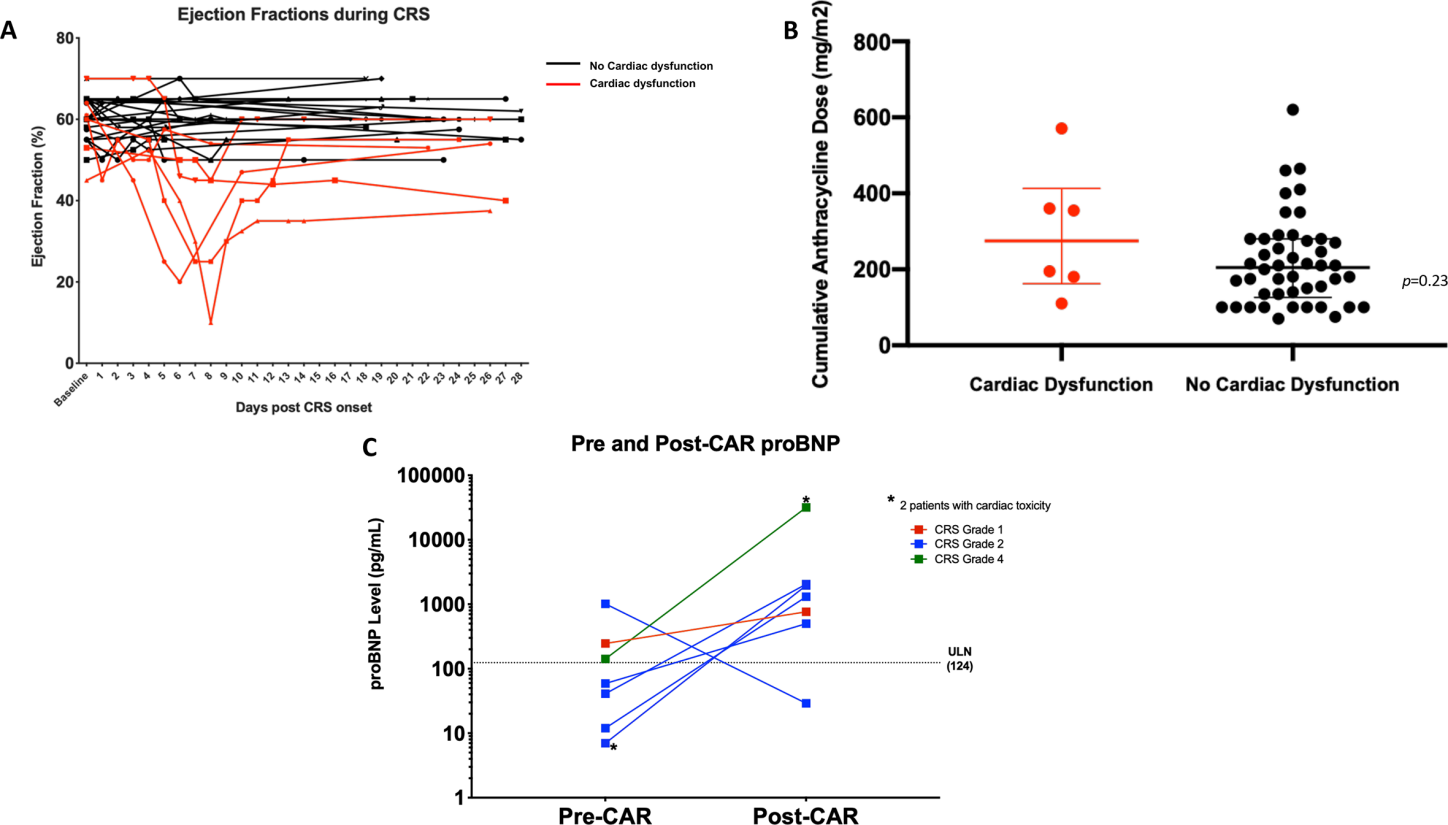
(A) Left ventricular EF (%) in all patients with CRS. Each line represents a patient’s EF during the course of CRS as determined by echocardiogram. Black lines indicate those patients with CRS who had preserved EF; without cardiac dysfunction. Red lines indicate those patients with CRS who had decreased EF and experienced cardiac dysfunction. (B) Cumulative anthracycline dose for all patients treated on CD19-28ζ CAR T-cell trial stratified by those who had decreased EF (min: 100 mg/m^2^, max: 570 mg/m^2^) and those that had preserved EF (min: 70 mg/m^2^, max: 620 mg/m^2^) as compared with their baseline echocardiograms. (C) Pre-CAR and Post-CAR T-cell therapy proBNP levels in seven patients. Two patients with the highest post-therapy pro-BNP levels (depicted by the *) had decreased EF and cardiac dysfunction. CAR, chimeric antigen receptor; CRS, cytokine release syndrome; pro-BNP, pro-B-type natriuretic peptide.

Six of 37 patients with CRS (16%) developed cardiac dysfunction, all of which occurred during the clinical course of CRS (table 3 and figure 1A) and all of whom required transfer to the ICU. Among nine patients with >grade 3 CRS, 4 (44%) developed cardiac dysfunction. There was a greater likelihood of patients with cardiac dysfunction developing CRS earlier (median 2.5 days post CAR T-cell infusion, p=0.015), having severe CRS (>grade 3, p=0.022) and receiving tocilizumab (p*=*0.006), but there was no association between development of cardiac dysfunction and administration of steroids (p*=*0.11). Those that developed cardiac dysfunction on trial had a significantly lower baseline GLS (p*=*0.040) as compared with those that did not develop cardiac dysfunction, however, there was no significant difference seen in baseline LVEF (p=0.59) between the two groups (table 1). No patients on study developed a clinically relevant arrhythmia. However, on day 4 of CRS, Patient 14 developed cardiac arrest but recovered following aggressive management, including temporary mechanical circulatory support (intra-aortic balloon pump).^6^ This patient received tocilizumab and steroids for grade 4 CRS. This patient’s cumulative lifetime anthracycline dose was 360 mg/m2, and he had a baseline LVEF of 64%, with substantial decrease in LVEF, to a nadir of 20% on day 5 of CRS. Peak troponin level (6.23 ng/mL) was observed on day 4 of CRS.

Resolution of cardiac dysfunction was observed in four of the six patients by day 28 post-CAR T-cell infusion, including patient 14. Two patients had persistent cardiac dysfunction with decreased LVEF at day 28. One of these patients had the lowest LVEF (10%) during CRS and the other had a modest decrease in LVEF from baseline (50%–40%); however, both patients recovered to baseline by the 3 months time point. Additionally, these two patients had among the highest baseline anthracycline exposure, 570 mg/m^2^ and 355 mg/m^2,^ respectively (figure 1B).

In 16 of 37 patients, echocardiograms performed during CRS had appropriate image views available to retrospectively analyze LV GLS. These echocardiograms were performed at a median of 5.5 days post-CAR infusion (range 2–12), and all 16 patients had abnormal LV GLS, with a median of 14.1% (range: 5.3%–17.9%). 14/16 patients with CRS had pre-GLS and post-GLS measurements (two patients did not have baseline LV GLS available for comparison) and 64.2% (9/14) had relative reduction in GLS >15%. This included three patients with normal strain at baseline, who developed abnormalities post CAR T-cell infusion. All six patients that developed cardiac dysfunction (defined by decreased EF) during CRS had concurrent abnormal myocardial strain, with a median LV GLS of 10.1% (range 5.3%–14.1%). Tables incorporating outcomes stratified by those patients with a significant relative decline in GLS >15% (6 additional patients) has been added as online supplementary file.

10.1136/jitc-2020-001159.supp1 Supplementary data

Thirteen patients had real-time conventional troponin assays measured during their CRS course as clinically indicated. Of the patients that were tested, 30.7% (4/13) had abnormal troponin, and all four of these patients had cardiac dysfunction measured by decreased LVEF. There was a limited cohort of patients (n=7) with proBNP monitoring, at baseline and serially after CAR T-cell infusion, with peak pro-BNP recorded as post-CAR time point. In this cohort, pro-BNP levels were elevated during CRS, with the highest elevations observed in two patients with cardiac dysfunction that developed during CRS (figure 1C).

## Discussion

As the indications for CAR T-cell therapy broaden, understanding the full spectrum of the toxicity profile is critical in order to safely treat patients. CRS, the main toxicity of CAR T-cell therapy, is associated with a host of cardiac manifestations which may include tachycardia, decrease in EF and hypotension, among others.^15 21^ We report on the first pediatric study evaluating cardiotoxicity and its relationship to CRS in children and young adults who received a CD19-28ζ CAR construct.^3 6 8^

Notably, patients referred to this study were heavily pretreated, having received a median of 4 chemotherapy cycles, with all patients exposed to anthracycline-containing regimens, and 44% of patients having had a prior HSCT and TBI. It is hypothesized that patients who have a significant history of anthracycline exposure or TBI may have inadequate cardiac reserve to sustain cardiovascular insults like those associated with CRS.^18^ LV strain measurements are emerging as a powerful tool in screening and monitoring patients undergoing potentially cardiotoxic therapies. Baseline and longitudinal abnormalities in GLS are used to detect subclinical LV dysfunction and may have additive value over EF and other imaging and clinical parameters.^29^ Among our baseline echocardiograms, only 6% (3/52) had abnormal EFs. In contrast, a substantial fraction of patients, 78% (29/37), had baseline abnormal GLS, reflective of possible cardiac toxicity from previous anthracycline exposure or irradiation history. While the subgroup of six patients developing cardiac dysfunction was too small to demonstrate predictive value of GLS, closer imaging surveillance in high-risk patients may allow earlier detection of cardiac abnormalities. Indeed, when retrospective analysis of pre-GLS and post-GLS values were performed, an additional six patients were identified as having subclinical LV cardiac dysfunction (online supplementary tables). Thus, prospective and real-time GLS monitoring preinfusion and postinfusion may provide valuable information and potentially earlier identification of acute and subacute cardiac strain that may impact a patients’ toxicity profile and the treatment thereof.

Although a majority of patients developed some cardiac toxicity on this study, the most common manifestation was sinus tachycardia observed in 36 of 37 patients with CRS. More significant cardiac dysfunction defined by decreased EF occurred in 6 of 37 (16%) patients who developed CRS (and in 6 of 52 (12%) of all treated patients). No patients on this study developed cardiac dysfunction independent of CRS. This is in line with the experience described by Burstein *et al*,^21^ who retrospectively demonstrated new systolic dysfunction in 10% of CAR T-cell treated children. In our high-risk patient population with refractory disease, our data suggest that cardiovascular toxicity associated with CAR T-cell therapy in children and young adults is both limited and reversible. Recognizing that more follow-up is needed, long-term dysfunction was not seen in our study or other recent reports.^17 18 21^

While shock associated with CRS is primarily vasodilatory in nature, due to capillary leak, patients may only be transiently responsive to fluid resuscitation, thus necessitating vasoactive mediations. Furthermore, a mixed vasodilatory and cardiogenic shock picture may be seen, thus necessitating additional inotropic support. In this study, 17% of all patients treated with CAR T-cells developed hypotension-requiring pressor medication, with only one patient requiring inodilator support with milrinone.

Utilization of biomarkers was obtained in this study in real time in a limited cohort of patients. Provider practice differences led to varying cases and times in which biomarkers were obtained. Serial proBNP levels measured pre-CAR and post-CAR T-cell infusion demonstrated proBNP elevation during CRS, with two of six patients who had decreased LVEF having peak elevations in pro-BNP during CRS. Additionally, myocardial injury indicated by troponin elevation was noted in 4 of 13 patients, all of whom had significant cardiac dysfunction defined by depressed LVEF. Although the number of patients tested was small, performing serial evaluations of these biomarkers was feasible and may be helpful in identifying high risk patients who may benefit from earlier medical intervention during CRS. Indeed, a recently published study in adult oncology patients demonstrated that cardiac injury and cardiovascular events were common post-CAR T-cell therapy, with 95% of their events occurring after an elevated troponin.^17^

In our cohort, severe CRS was associated with a higher likelihood of developing cardiac dysfunction. Recent data supports the utilization of early intervention with tocilizumab and/or steroids to reduce the severity of CRS in an effort to improve outcomes and toxicity profiles, without impacting antileukemia activity.^30^ Furthermore, the aforementioned study in adults receiving CAR T-cell therapy found that a delay in administration of tocilizumab for CRS was associated with a 1.7-fold increase in cardiovascular events.^17^ Thus we anticipate as pre-emptive CRS mitigation strategies are more readily employed, impact of CRS on cardiac dysfunction may be lessened.

Limitations to this study include the largely retrospective nature, especially with regard to analysis of the first 13 patients as comprehensive cardiac monitoring (eg, formal echocardiograms) of patients who went to the ICU was not incorporated until after patient 14. Thus, some patients earlier in the study may have developed transient cardiac dysfunction, which was not recognized. While it is highly unlikely that severe cardiac dysfunction would go unnoticed, the number of patients with any cardiac toxicity may be underrepresented. Additionally, cardiac workup was subject to provider discretion. Although some real-time evaluations were performed during the course of the study, frequency of studies and medications given were at the discretion of the treating physician. Another limitation to our study is that pretherapy and post-therapy pro-BNPs were not routinely performed on all patients. Treating physicians were more likely to obtain serial proBNPs on those who had cardiac dysfunction defined by echocardiogram, thus limiting our ability to perform multivariate analysis and make definitive conclusions regarding these results and the applicability to the broader CAR T-cell population. Also, the use of conventional troponin assays, as compared with high-sensitivity troponin assays, measuring biomarkers may have missed patients with subtle cardiac stress. However, the sensitivity of the conventional troponin assay is adequate to detect myocardial injury in those with clinically meaningful depressed LVEF such as patient 45, who had serial troponin levels measured during the course of CRS, all of which were undetectable. Finally, this was a single institution study, with a small number of patients, thereby limiting the power of our findings. Future steps include establishing a CAR T-cell consortium with multiple institutions who treat pediatric and young adult patients in order to systematically collect and analyze toxicity data and centralize such results in an effort to better identify patterns of late toxicities.

**Figure 2:**
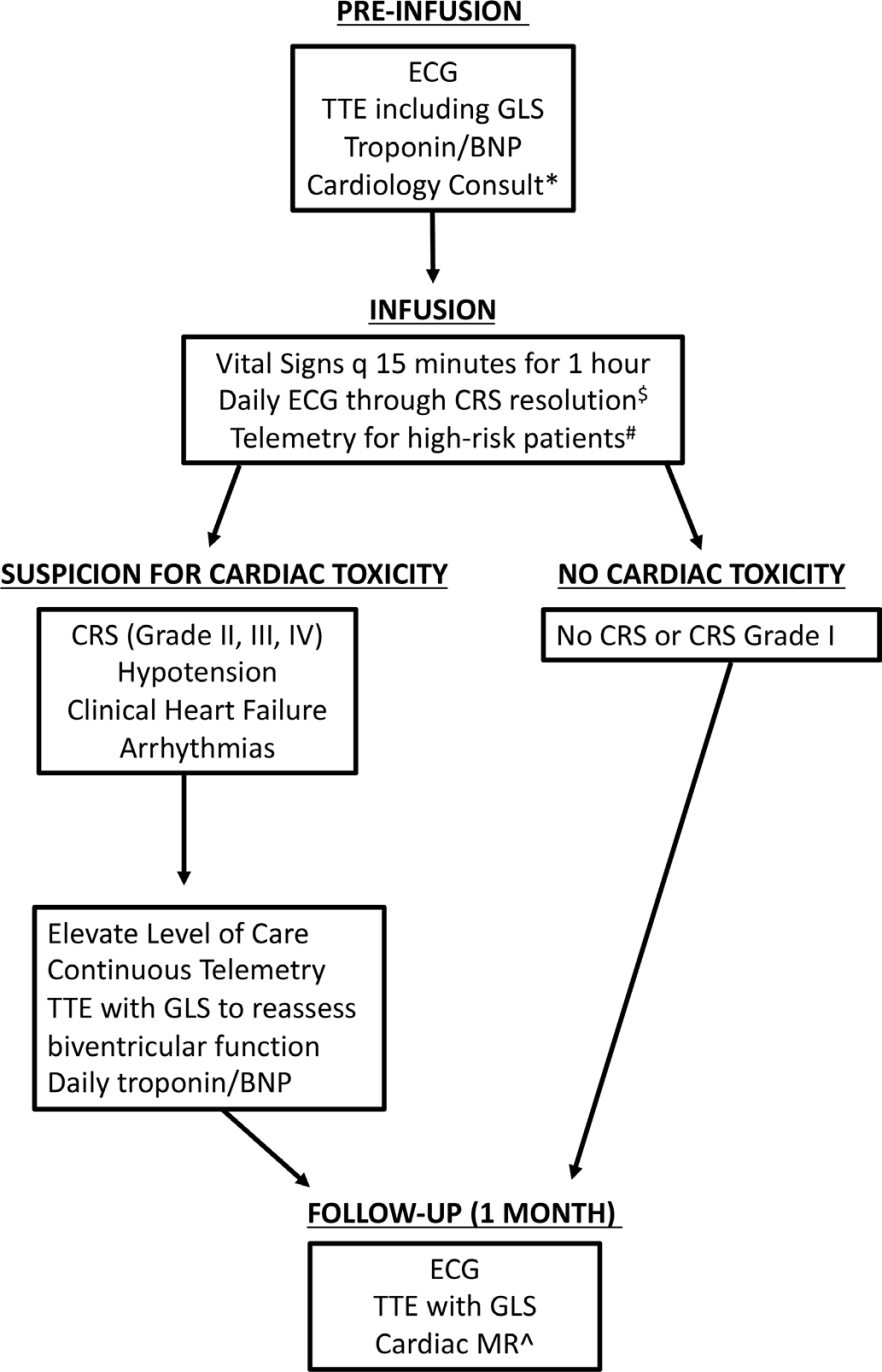
Cardiac screening and monitoring algorithm for pediatric and young adult patients undergoing CAR T-Cell Therapy. *Obtain cardiology consult in patients with a history of heart failure (preserved or reduced ejection fraction (EF), known cardiomyopathy, cardiac arrhythmias, prior chest/mediastinum radiotherapy, baseline BNP or troponin elevated above upper limit of normal. Consider cardiology consult in patients with asymptomatic LV dysfunction (LVEF <53% or abnormal GLS) or cumulative anthracycline dose >300 mg/m^2^; ^$^Monitor for conduction abnormalities and QT prolongation; ^#^High-risk patients include those that required cardiology consult prior to infusion; ^∧^Consider cardiac MR in patients with new or worsened LV dysfunction (LVEF <53% or drop in GLS >15% from baseline) that has persisted after CAR T-cell therapy. BNP, B-type natriuretic peptide; CAR, chimeric antigen receptor; CRS, cytokine release syndrome; GLS, global longitudinal strain; TTE, transthoracic echocardiogram.

There is currently no systematic guidance on how to monitor for post CAR T-cell cardiac toxicity, or provisions for how long the duration of monitoring should continue in children. The findings in this study support the need for a systematic approach for the management of CRS-associated cardiotoxicity that incorporates close clinical surveillance. We provide an algorithm (figure 2) to systematically and prospectively monitor pediatric patients receiving CAR T-cells for cardiac toxicity, with monitoring of high-risk patients until resolution of the aforementioned toxicities to baseline. This guidance incorporates cardiac enzymes, EKGs and echocardiograms at various time points during the course of CAR therapy with the hope of broad implementation that will facilitate cross-trial comparisons of cardiac toxicity. These studies should allow for rapid recognition of cardiac side effects in patients with CRS and allow for timely and optimal therapy. To this end, early consultation with cardiologists and ICU providers to help with management of patients, especially with hemodynamic support, is imperative.

In conclusion, this study of patients treated with CD19-28ζ CAR T-cells demonstrates that cardiotoxicity associated with CRS is limited and generally reversible with severe cardiac dysfunction occurring less frequently. A systematic approach of clinical surveillance and comprehensive cardiac evaluations would facilitate earlier identification of patients at high risk of developing severe cardiac dysfunction.
