# Supplementary material for: Impact of cytokine release syndrome on cardiac function following CD19 CAR-T cell therapy in children and young adults with hematological malignancies
Source: J Immunother Cancer. 2020 Sep 3;8(2):e001159. doi: 10.1136/jitc-2020-001159 (PMC7473612; doi:10.1136/jitc-2020-001159)
Supplement: Supplementary data [file jitc-2020-001159supp001.pdf]

**Supplemental Table 1.**
**Patient Demographics Stratified by those that had Cardiac Dysfunction defined by > 15% Relative Change in Global Longitudinal Strain and/or ≥ 10% Absolute Decrease in Left Ventricular Ejection Fraction (LVEF) Compared with Baseline**

| | | **Cardiac Dysfunction n=12** | **No Cardiac Dysfunction n=40** | ***p* Value*** |
|---|---|---|---|---|
| Age, median (range), y | | 15.3 (10-30) | 12.8 (4-27) | 0.092 |
| Male, n (%) | | 11 (91.7%) | 30 (75%) | 0.42 |
| Diagnosis | ALL, n (%) | 11 (91.7%) | 39 (97.5%) | 0.41 |
| | NHL, n (%) | 1 (8.3%) | 1 (2.5%) | |
| Primary refractory | | 4 (33.3%) | 9 (22.5%) | 0.096 |
| Prior lines of therapy | >4, n (%) | 0 (0%) | 9 (22.5%) | |
| | 2-4, n (%) | 12 (100%) | 31 (77.5%) | |
| Prior HSCT | 0, n (%) | 6 (50%) | 23 (56.5%) | 0.74 |
| | 1, n (%) | 6 (50%) | 12 (57.5%) | |
| | 2, n (%) | 0 (0%) | 5 (12.5%) | |
| Prior TBI, n (%) | | 6 (50%) | 17 (42.5%) | 0.74 |
| Prior immunotherapy, n (%) | | 2 (16.7%) | 9 (22.5%) | 1.00 |
| Prior anthracycline exposure, median (range), doxorubicin equivalents | | 202 (110-571) | 205 (70-620) | 0.69 |
| Baseline left ventricular ejection fraction, median (range), % | | 61% (50-70%) | 60% (50-72%) | 0.77 |
| Baseline left ventricular global strain, median (range),% n=37 | | 18.2% (14.1 to 22.2 %) | 16.8% (14.1 to 23.5 %) | 0.78 |
| Performance status, median (range) % | | 90% (40-100%) | 90% (50-100%) | 0.11 |

Cardiac dysfunction is defined as a ≥ absolute 10% decrease in left ventricular ejection fraction (LVEF) compared with baseline, new onset left ventricular systolic dysfunction ≥ grade 2, LVEF < 50%, or >15% relative reduction in left ventricular global longitudinal strain (GLS) compared with baseline; Global longitudinal strain (GLS) is presented in absolute numbers |%|.

**Supplemental Table 2.**
**Characteristics of Patients with Cytokine Release Syndrome (CRS) Stratified by those that had Cardiac Dysfunction defined by > 15% Relative Change in Global Longitudinal Strain and/or ≥ 10% Absolute Decrease in Left Ventricular Ejection Fraction (LVEF) Compared with Baseline**

| | | **Cardiac Dysfunction n=12** | **No Cardiac Dysfunction n=25** | |
|---|---|---|---|---|
| Time to onset of CRS, median (range), d | | 3 (1-7) | 6 (1-12) | 0.052 |
| CRS Max Grade^ | 1, n (%) | 1 (8.3%) | 13 (52%) | 0.43** |
| | 2, n (%) | 7 (58.3%) | 7 (28%) | |
| | 3, n (%) | 2 (16.7%) | 4 (16%) | |
| | 4, n (%) | 2 (16.7%) | 1 (4%) | |

| | | | | |
|---|---|---|---|---|
| ASTCT CRS Max Grade[#] | 1, n (%) | 1 (8.3%) | 15 (60%) | 0.006** |
| | 2, n (%) | 3 (25%) | 6 (24%) | |
| | 3, n (%) | 6 (50%) | 3 (12%) | |
| | 4, n (%) | 2 (16.7%) | 1 (4%) | |
| Duration of fever > 38, median (range), d | | 5 (3-11) | 5 (1-14) | 0.10 |
| Duration of fever > 40, median (range), d | | 4 (1-6) | 2 (1-6) | 0.19 |
| Duration of tachycardia[@], median (range), d | | 7 (2-9) | 5 (1-30) | 0.75 |
| ICU transfer, n (%) | | 11 (91.6%) | 10 (40%) | 0.004 |
| Received Tocilizumab, n (%) | | 4 (33%) | 3 (12%) | 0.18 |
| Received steroids, n (%) | | 3 (25%) | 1 (4%) | 0.090 |
| Required vasopressor support | 1 agent | 4 (33%) | 2 (8%) | 0.11[$] |
| | >1 agent | 1 (8.3%) | 2 (8%) | |
| Required milrinone, n (%) | | 1 (8.3%) | 0 | 0.32 |
| Required mechanical ventilation, n (%) | | 3 (25%) | 1 (4%) | 0.090 |

Cardiac dysfunction is defined as a $\geq$ 10% absolute decrease in left ventricular ejection fraction (LVEF) compared with baseline, new onset left ventricular systolic dysfunction $\geq$ grade 2, LVEF < 50%, or >15% relative reduction in left ventricular global longitudinal strain (GLS) compared with baseline. Intensive care unit, ICU; ^CRS Max Grade as per Lee, et al. Blood 2014; [#]American Society for Transplantation and Cellular Therapy (ASTCT) CRS grading was retrospectively incorporated; [@]n=36; *The *p* value is comparing treatment course characteristics of those with and without cardiac dysfunction. **CRS grading compared mild CRS, grade 1/2 vs severe CRS, grade 3/4; [$]*p* value compared requiring any pressor support vs none in those with and without cardiac dysfunction.

**Supplemental Table 3:**
**Characteristics of Patients with Cardiac Dysfunction including those that had > 15% Relative Change in Global Longitudinal Strain**

| Patient | Prior Anthracycline Exposure (mg/m$^2$) | Baseline LVEF | Lowest LVEF | Baseline LVGLS | Lowest LVGLS | Max CRS ^ grade | Vasoactive support? | Troponin Elevation? | Peak Troponin (ng/mL) |
|---|---|---|---|---|---|---|---|---|---|
| 14 | 360 | 64% | 20% | Not performed[$] | 7.6% | 4 | Yes | Yes | 6.23 |
| 16 | 110 | 70% | 45% | 18.7% | 14.1% | 3 | No | Yes | 0.117 |
| 22 | 210 | 55% | 50% | 15.9% | 10.3% | 2 | Yes | Not performed | -- |
| 25 | 238 | 58% | 65% | 18.8% | 12.1% | 2 | No | Not performed | -- |
| 32 | 270 | 65% | 60% | 19.5% | 15.2% | 2 | No | Not performed | -- |
| 38 | 100 | 65% | 60% | 22.2% | 14.3% | 1 | No | Not performed | -- |

| | | | | | | | | | |
|---|---|---|---|---|---|---|---|---|---|
| 39 | 195 | 60% | 25% | 14.6% | 5.9% | 3 | No | Yes | 0.113 |
| 45 | 570 | 55% | 10% | 14.1% | 5.3% | 4 | Yes | No* | <0.010 |
| 46 | 181 | 60% | 64% | 21.8% | 17.9% | 2 | No | No* | <0.010 |
| 49 | 100 | 65% | 65% | 17.8% | 13.9% | 2 | Yes | Not performed | -- |
| 51 | 355 | 50% | 40% | 11.6% | 12.5% | 2 | No | No* | <0.010 |
| 52 | 180 | 61% | 45% | Not performed[#] | 14% | 2 | Yes | Yes | 0.016 |

Cardiac dysfunction is defined as a ≥ 10% absolute decrease in left ventricular ejection fraction (LVEF) compared with baseline, new onset left ventricular systolic dysfunction ≥ grade 2, LVEF < 50%, or >15% relative reduction in left ventricular global longitudinal strain (GLS) compared with baseline; Global longitudinal strain (GLS) is presented in absolute numbers |%| [$]Outside baseline echo; [#]GLS was not able to be performed due to image quality on baseline echo; ^ CRS Max Grade as per Lee, et al. Blood 2014. *Troponin levels were drawn and undetectable, lower limit of detection 0.010 ng/mL
